# The *Toxoplasma* Kinase ROP18: An Active Member of a Degenerate Family

**DOI:** 10.1371/journal.ppat.0030016

**Published:** 2007-02-16

**Authors:** Anthony P Sinai

**Affiliations:** The Scripps Research Institute, United States of America

Intracellular parasitism defines the most intimate of interactions between a pathogen and host. Inherent in this equation are the needs of the pathogen to enter the cell and establish a replication-permissive niche while neutralizing and subverting cellular defenses. The protozoan parasite Toxoplasma gondii is particularly adept at every stage of intracellular parasitism, which has contributed to its being among the most cosmopolitan parasites on the planet [1]. *Toxoplasma* is a member of the Apicomplexa, so noted by the presence of specialized cytoskeletal and secretory organelles (the apical complex) that play a critical role in invasion [2]. Most prominent among these secretory organelles are the club-shaped rhoptries [3,4]. Recent work, including a detailed cataloging of the rhoptry proteome [5], coupled with the availability of the T. gondii genome and gene expression data (http://www.ToxoDB.org; [6]), have resulted in new insights into rhoptry functions. Among the activities inferred from the analysis of the proteomic and genomic data was a family of predicted serine threonine kinases related to the rhoptry protein ROP2 [7]. While ROP2 and several other members of the ROP2 family retain “molecular fossils,” reflecting their ancestry as kinases, they do not possess kinase activity on account of degeneracy and deletions at key catalytic sites [7]. However, bioinfomatic and structural prediction studies reveal that two members, ROP16 and ROP18, do retain all the elements needed to classify them as true kinases [7].

The focus of this Opinion is the rhoptry-derived kinase ROP18, the initial biochemical analysis of which appears in this issue of *PLoS Pathogens* from the laboratory of Jean François Dubremetz [8]. This characterization is particularly timely given a pair of recent papers from the Boothroyd [9] and Sibley [10] laboratories identifying ROP18 as a critical contributor to virulence, a finding that represents the first virulence factor identified in *Toxoplasma* using classical forward genetic approaches. I hope in this article to integrate the results in these seminal papers and present them in the context of *Toxoplasma* biology, virulence, and microbial pathogenesis in general.

Rhoptries are unique secretory/excretory organelles found exclusively in the Apicomplexa that are discharged at the time of invasion and are critical in the establishment of a productive infection [11,12]. Functional studies emanating from the analysis of the rhoptry proteome [5] identified factors critical in establishing the machinery for both parasite invasion and the establishment of the nascent parasitophorous vacuole [13,14]. The parasitophorous vacuole, which defines the replication-permissive niche, is delimited from the host cytoplasm by the parasitophorous vacuole membrane (PVM), a remarkable and enigmatic organelle in its own right [15]. To date, all proteins secreted from the rhoptry either associate with the PVM or are transported across it into the host cell cytoplasm [5,16]. Among these members of the ROP2 family are several particularly interesting proteins. ROP2, the founding member of this family, is secreted to the PVM at the time of parasite invasion, where it is believed to establish itself as an integral membrane protein with its N-terminus exposed to the host cell cytoplasm [17]. The mechanism for membrane association, however, is controversial in light of recent molecular modeling analyses [7]. The presence of an N-terminal signal on the secreted protein that is reminiscent of a canonical mitochondrial import signal has implicated ROP2 as the mediator of the high affinity binding of mitochondria to the PVM [18,19]. The availability of the T. gondii genome led the Dubremetz group to extend the ROP2 family to 16 members based upon both bioinformatic criteria and functional proteomic data [7]. A somewhat surprising revelation regarding the provenance of ROP2 family suggests they have all evolved from a progenitor serine threonine kinase on the basis of the presence of key signature motifs [7]. These motifs, however, have degenerated, particularly at the N-terminus, where critical structural and sequence components for kinase activity are either missing of significantly mutated in most of the members [7]. Most of these degenerate ROP2 family members are predicted [7] or have been demonstrated [20] to lack detectable kinase activity. Interestingly, in most of these proteins lacking kinase activity, the key structural element, a kinase fold at the C-terminus, remains unchanged, which suggests a strong selective pressure to retain it [7]. This fold in ROP2, and the other closely related proteins, appear to be transmembrane domains [17], which may suggest that a divergence of function has accompanied the evolution of this family. What makes ROP18 and ROP16 stand out in this family is that they retain all of the bioinformatic and structural elements required to designate them as putative functional kinases [7].

There is a significant difference in the in silico prediction of an enzymatic activity and its biochemical demonstration. The studies described in this issue of the journal provide the first biochemical and functional data that define ROP18 as a bona fide kinase [8]. ROP18 is secreted to the PVM [8], a location where an impact on parasite, host, or both parasite and host factors can be predicted. While the identity of the substrate for ROP18 remains elusive, it has been observed that mature recombinant ROP18 selectively phosphorylates an as yet unknown 70-kDa parasite protein, but not proteins in host cell extracts [8]. This activity, however, is lost with an engineered mutant with a critical active site mutation (D394A) [8]. Together, these data represent the first functional biochemical validation of a rhoptry-derived kinase. The authors report on multiple but futile attempts to knock out ROP18, suggesting its activity may be essential. The importance of ROP18 emerges in the observation that the overexpression of the protein results in a dramatic increase in parasite proliferation [8], an observation identical to that made by Taylor et al. [10]. Critically, the same mutation resulting in the loss of kinase activity (D394A) for ROP18 also results in the loss of the accelerated growth phenotype [8]. Furthermore, the observation that the enhanced growth is restricted to the vacuole overexpressing ROP18 in a doubly infected cell argues for a localized effect that is unlikely to directly impact changes within the infected cell, in effect trans-complementing other wild-type vacuoles [8]. This is consistent with the apparent failure of recombinant ROP18 to phosphorylate any host cell proteins [8]. With its localization at the PVM, one might speculate about how this kinase may impact parasite growth. The PVM, as the interface between the parasite and the host, serves as a signaling platform as well as the site for nutrient uptake [15]. One may speculate that ROP18 is involved in phosphorylating a substrate protein(s), thereby increasing the efficiency for the uptake of a limiting nutrient. Whether the 70-kDa protein in tachyzoite extracts that is phosphorylated by the recombinant enzyme is secreted to the PVM, however, remains an open question. What is known, however, is that another ROP2 family member, ROP4, serves as a substrate for an as yet unknown kinase and is phosphorylated only in infected cells [20].

The recent in silico structural analyses of the ROP2 family have brought into question whether a domain that is critical for kinase activity and annotated as a transmembrane domain for ROP2 is in fact one [7]. If not, the mechanism for anchoring ROP18 (and other ROP2 members) at the PVM presents a dilemma complicated by the fact that ROP18 does in fact have kinase activity [7]. In the PLoS paper by Dubremetz and colleagues, an experiment designed to examine the impact of expression of mature (like most other ROP2 family members, ROP18 is proteolytically processed en route to the rhoptry [7]) ROP18 in mammalian cells presented a curious finding. ROP18 expressed in cells was found to have no specific localization; however, infection of such a cell resulted in the decoration of host cytoplasm–expressed ROP18 at the PVM, suggesting a very specific interaction [8]. It is possible, therefore, that ROP18 is in fact secreted into the host cytoplasm and is bound by a specific parasite adaptor molecule on the cytoplasmic aspect of the PVM, thereby allowing the kinase fold designated for ROP2 as a transmembrane domain [17] to serve its enzymatic function.

What makes all these activities associated with ROP18 particularly relevant are the recent publications from the Boothroyd [9,21] and Sibley [10] laboratories identifying ROP18 as an important virulence determinant. To understand the importance of this work, a short primer on T. gondii pathogenesis and virulence is presented.

T. gondii is a truly cosmopolitan parasite, with a host range encompassing birds, terrestrial mammals, and marine mammals [1]. Felines serve as the definitive host, as it is only in their gastrointestinal tracts that the sexual cycle of the parasite is manifest, resulting in the shedding of the environmentally stable and highly infectious oocysts [1]. T. gondii has evolved an additional means of transmission mediated by tissue cysts in the muscles and organs of infected intermediate hosts [1]. This transmission cycle by carnivory has contributed to the explosion of the host range while serving as a bottleneck for genetic diversity [22]. Despite is ubiquitous host range, the population structure of the parasite is remarkably narrow, with genetic analyses revealing a largely clonal population structure, although a pool of divergent “exotic” organisms persists [23–25]. Despite their high homology, profound differences in acute virulence are noted between strains. Accordingly, the hypervirulent Type I strains are universally lethal in mice, with a single parasite serving as the LD_100_ [25]. In contrast, the “avirulent” Type II and III strains are much attenuated with an LD_50_ > 1000 organisms, a minimum 3 log difference [25]. Virulence is a complex phenotype governed by factors including efficiency of parasite growth, resistance to host defenses, and the capacity to disseminate within the host. Among these properties, rapid parasite growth and the immune and physiological consequences of the increasing systemic parasite burden serve as the strongest correlate to lethality in vivo [26].

Performing genetic crosses of *Toxoplasma* is not trivial, as they require the use of cats and the handling of large concentrations of the highly infectious oocysts [27]. In the papers from the Boothroyd and Sibley, group crosses were conducted between the Type I × Type III (hypervirulent × avirulent) [10] and Type II × Type III (avirulent × avirulent) [9] and the progeny examined for diverse virulence traits, including mortality at defined infective doses [9,10], intracellular growth [10], migration (in soft agar and transmigration of epithelia) [10], induction of host transcriptional responses [9,21] and immune parameters, including serum responses [10], and the capacity to induce Th1 cytokines [9]. Mapping of the virulence-associated traits by quantitative trait locus analysis revealed several loci across the genome associated with the differences in the traits listed above. Quantitative trait locus analysis, mapping by which is dependent on genetic recombination, lacks the sensitivity to pinpoint the responsible locus or gene. A quantitative trait locus on chromosome VIIa drew particular attention, as its impact was felt on multiple virulence-associated phenotypes. Examination of this region (1.1 Mb with 140 predicted genes) drew attention to one specific locus on account of its predicted function, predicted localization of the protein, and high density of polymorphisms between the different clonotypes across the virulent (Type I) and avirulent (Types II and II) parasites [9,10]. Confirmation of the impact of strain-specific polymorphisms at this locus on the virulence phenotypes by the cross expression of strain-specific alleles in the distinct genetic backgrounds reconfirmed its importance as the key mediator responsible for the observed phenotypic difference [9,10]. This locus turned out to be ROP18 [9,10], the biochemical and functional characterization of which is presented in this issue of *PLoS Pathogens* in the paper from the Dubremetz group [8].

While a blockbuster in its own right, ROP18 turns out not to be the only star of the ROP2 family (notwithstanding ROP2 itself, for which I have a personal affection). A locus on chromosome VIIb was also found to be involved in the strain-specific (TypeII versus TypeIII) differences in the capacity to alter host transcriptional responses using genetic mapping approaches [21]. This locus, ROP16, is also predicted to be an active kinase [7,21], but, unlike ROP18, which remains at the PVM [7,10], is translocated into the host cell nucleus [21]; this is a property noted for a T. gondii–encoded protein, phosphatase 2C [16]. This protein's localization belies its apparent function of impacting the host transcriptional responses by subverting STAT3 and 6 signaling among the targets for which key cytokines are involved in the immune response to T. gondii [21]. Our own work reveals that T. gondii subverts the transcriptional responses regulated by NFκB using a parasite-encoded, PVM-localized activity capable of phosphorylating the inhibitor of NFκB, IκBα [28,29]. We are in the process of identifying this activity, which does not appear to be rhoptry-localized, based on the kinetics of its appearance at the PVM [29]. This all suggests that kinases and likely other signaling activities such as phosphatases, regulatory adaptors, and enzymatic modifiers (ubiquitin ligases/de-ubiquitinating activities) delivered to the PVM play a central role in the establishment of a productive infection. An ongoing proteomic analysis (M. Martin, T. Liu, B. Lynn, and A. Sinai, unpublished data) reveals evidence for all of these activities at the PVM.

The exploitation of genomic and expression data, coupled with large-scale proteomic screens, has set the stage for the detailed dissection of complex phenotypes at the molecular level. While the evolution of the ROP18 story presents a new paradigm in microbial pathogenesis, many other important questions in the biology of the parasite and the parasite–host interaction remain wide open. 

## Abbreviations

PVM: parasitophorous vacuole membrane
